# Modern Sociology

**Published:** 1897-01-16

**Authors:** 


### Modern Sociology.

### THE BIGGEST WORKSHOP IN THE WORLD.

Strange to say, this is not located in America, where they have most things on a big scale, but in staid Germany, which we are only now beginning to recognise as a commercial rival to ourselves. The biggest workshop in the world is the iron and steel factory of Frederick Krupp, at Essen. For a thousand years or so Essen was a dull agricultural village. At the beginning of this century its population numbered some 3,000 inhabitants. In 1830 the population had risen to 5,457, of whom Herr Krupp employed ten. Things do not seem to have prospered with him at first, for next year he had only nine workmen. Still he persisted, and we find that in 1848 the number of workmen had risen to 72. The population of the village was then somewhere about 8,000. In 1894 the population of Essen was about 80,000, of whom 17,000 were employed in the Krupp works; in fact, Essen now exists by and for the Krupp firm. It is doubtful if there is a family in the town which does not derive its living directly or indirectly from these works. The Essen workmen are not, however, the only ones whom Krupp employs. The firm has ironworks elsewhere, besides three coal mines, and various iron mines in Germany and Spain. But it is with Essen that it is most intimately associated, and it is in Essen that it has carried out that system of paternal government with which its name is associated.

The immediate result of a speedy increase of the chief industry of any town or district is bad morally and physically. Coventry during the present cycling boom will bear witness to that. The first evil is overcrowding. In Essen, whereas in 1840 the average number of persons in each house was 7·53, in 1890 it had risen to 16·22. In the distinctively workmen's quarter the density was even greater, and in one street the average was nearly 24 persons to each house. The houses had indeed multiplied fivefold in the half century, but the population had increased more rapidly still. The overcrowding sprang from a sudden increase in rents, and resulted naturally in an increase of deaths. In 1855 the rent of a two-roomed house ran from £3 13s. to £4 4s., but soon rose to more than £7. So for economy's sake people crowded together and died. In Essen generally the death-rate was 3·41 per cent. In the workmen's streets it was 4·59. Another evil was the high price charged by the tradesmen who supplied the necessaries of life. The workmen were bribed with presents and cajoled with liquor, encouraged to get into debt in order that the merchant should have them more completely in his power, in short, robbed of a portion, great or small, of their earnings.

The Krupp firm took up the matter. They established co-operative stores, where the necessaries of life were retailed at a reasonable rate. But this was only the beginning of a series of institutions in which the all-powerful firm took upon itself the functions of a State. A system of insurance against sickness and accident, the encouragement of life insurance, old-age pensions, savings banks, were all instituted. Schools, primary, secondary, and technical, were established, the latter including provision not only for the training of the future workman, but for that of his wife, who is taught all the arts that go to the making of the complete "hausfrau." The Krupp workmen are attended in sickness by one of sixteen doctors. These doctors are appointed by the firm, but once appointed the income of each depends on his popularity, for the invalid may choose his own medical attendant, and each doctor is paid in proportion to the work he has done.

But the arrangements for housing the workers form probably the largest effort of Krupp benevolence. The firm wished to erect cottages, each one detached and standing in a garden, but this was found impossible. There was not land enough within reasonable distance of the works for all the houses needed, for in the years 1871 to 1873, when the problem was seriously taken in hand, houses were provided for 2,358 families. The cost of land, the cost of transporting water, the cost of journeying to and from the works, combined to put such a scheme out of the question. Therefore the town of Essen is largely a town of tenements. Each tenement, however, is self-contained; though all the tenements in a house have a common outside entrance, they are in all other respects completely isolated. The blocks are broken up by streets and numerous open spaces, there is abundance of water from the Krupp water-works, good gas from the Krupp gas-works; if not æsthetic, the dwellings are comfortable and sanitary. The houses are of various kinds, according to the classes of workmen who are meant to be accommodated and their wages. They range from two-room tenements, the rents of which vary from £3 to £5 5s., up to semi-detached or detached houses of five rooms or more, which are rented at various prices, according to their accommodation and convenience. The lowest rent for one of these is £10, and the highest about £16 10s. Each block or house possesses a cellar, a garden, and a drying-green. In speaking of these houses as an effort of benevolence, it was not meant that they were given in charity. They return a profit on the capital expended.

It is regarded by some working men as an inconvenience to live in a house provided by the master, because if they leave his service they must vacate it. A widow, even if she can afford to live in her old home, must make way for the next worker. To meet this difficulty the Krupp firm, though they do not sell any of the houses they have built, have set aside a sum to be lent to workmen of approved thrift and industry who wish to build homes for themselves. Three per cent. interest is charged on the loan, which is expected to be paid off in monthly instalments, though in case of illness the repayments may be suspended for a time.

These arrangements are all meant for married men, but for the unmarried there are provided large boarding-houses, each accommodating two hundred inmates, where board, lodging, and washing, besides the use of a library, a billiard-table, and a bowling-alley, are provided for the sum of ninepence-halfpenny a day. There is also a co-operative boarding-house for workmen of a higher rank. This is meant to hold thirty men; and while the rent of rooms is fixed according to the scale of comfort in them, all other expenses depend on the scale of comfort agreed on by the inmates, who elect from their number a general manager, and commit the conduct of the house to him. The share of the cost to each individual, including rent and a very liberal dietary, is a fraction over eighteenpence a day.

The Krupp firm has spent much in both thought and money for the well-being of its employés; but the partners declare emphatically that they have been rewarded by getting a higher class of workmen, and being remarkably free from labour difficulties.

